# Health Literacy Influences Men's Active and Passive Cancer Information Seeking

**DOI:** 10.3928/24748307-20190430-01

**Published:** 2019-07-19

**Authors:** Frances J. Drummond, Mary Reidy, Christian von Wagner, Vicki Livingstone, Jonathan Drennan, Mike Murphy, Colin Fowler, Mohamad M. Saab, Mairin O'Mahony, Josephine Hegarty

## Abstract

**Background:**

For cancer prevention information to be effective, it must be accessible to its target populations. Prevalence of inadequate health literacy (HL) is high, but there is a dearth of information on the impact of HL on men's cancer information seeking.

**Objective:**

We investigated (1) men's cancer information seeking behaviors, (2) the effect of HL on men's cancer information seeking behavior, and (3) men's preferences for cancer information, considering their HL level. From a national perspective, we investigated men's information seeking behavior from the Irish Cancer Society (ICS), the largest provider of cancer information in Ireland.

**Methods:**

Men from adult literacy classes and men's groups were invited to complete a questionnaire. General and ICS-specific cancer information seeking behavior was investigated. Univariate and multivariate logistic regression models were conducted with “ever” seeking cancer information from any source, and actively seeking and passively acquiring ICS information as dependent variables.

**Key Results:**

Overall, 259 men completed the questionnaire and 44% had inadequate HL. About one-half of responders reported “ever” actively looking for cancer information. In the study group, 19% actively sought and 67% passively acquired ICS-specific information. In multivariate analysis, the odds of actively seeking (2.93; 95% CI [1.05, 8.15]) or passively acquiring (4.7; 95% CI [1.99, 11.05]) ICS-specific cancer information was significantly higher among those with adequate versus inadequate HL, respectively. HL was not significantly associated with odds of “ever” cancer information seeking in multivariate analysis (odds ratio 1.81; 95% CI [0.90, 3.63]). Men want information about cancer prevention. Suggested future cancer information sources differed by HL levels. General practitioners and the Internet were the preferred source for men with inadequate (53.3%) and adequate HL (57%), respectively.

**Conclusions:**

Men both passively acquire and actively seek cancer prevention information. Multimodal dissemination of cancer prevention information is necessary to reach a wide cross-section of men, including those with inadequate HL. This could potentially lower men's cancer burden and reduce gender inequalities in cancer mortality. **[*HLRP: Health Literacy Research and Practice*. 2019;3(3):e147–e160.]**

**Plain Language Summary:**

Most men get cancer prevention information by coming across it passively in their daily lives, instead of actively looking for this information. Men with low health literacy are less likely to obtain cancer information both passively and actively. Men want this information. Organizations need to make this information available in many places and formats (e.g., Internet, doctor, television, sports clubs).

An estimated 30% to 50% of cancer cases are preventable, and this rate is higher among men than women (Danaei, Vander Hoorn, Lopez, Murray, & Ezzati, 2005; Ott, Ullrich, Mascarenhas, & Stevens, 2011). Despite this, cancer remains one of the leading causes of mortality and morbidity, with incidence and mortality higher in men (Ferlay et al., 2013). International and national organizations develop information programs to raise awareness of cancer risks and prevention strategies. The effectiveness of these is measured by the extent of information spread, understanding, and impact on the behavior of target populations. However, health information is constantly evolving, and with the growth of the Internet there has been a huge proliferation in the volume of information. This makes it difficult for people to keep abreast of changes and to find information applicable to them. Additionally, inadequate health literacy is at “epidemic” levels (Davis & Wolf, 2004; Marshall, Sahm, & McCarthy, 2012)

Health literacy is multidimensional. An integrative conceptual model states:

HL is linked to literacy and entails people's knowledge, motivation and competencies to access, understand, appraise and apply health information to make judgements and take decisions in everyday life concerning healthcare, disease prevention and health promotion to maintain or improve quality of life

(Sørensen et al., 2012 [p. 3]).

People with inadequate health literacy are more likely to be older, male, from minority groups, and have lower education (Kumar et al., 2017; MacLeod et al., 2017). Inadequate health literacy is associated with many negative health consequences, including lower disease knowledge, lower use of preventive services, higher mortality rates, and higher health care costs (Cutilli, Simko, Colbert, & Bennett, 2018; Mantwill, Monestel-Umana, & Schulz, 2015).

Health literacy is a lifelong process. One of the first steps is the ability to seek, find, and obtain health information. Health information is commonly obtained by two methods: (1) active information seeking, which is the process of looking for and amassing information (Niederdeppe et al., 2007), and (2) information scanning, a passive process in which a person encounters information (Lee, Zhao, & Pena-y-Lillo, 2016) and decides on the attention given to it.

Despite the prevalence and the negative health impact of inadequate health literacy, recent systematic reviews have highlighted the dearth of research on the effect of health literacy on men's cancer information seeking behavior (Saab et al., 2018) and in intervention design (Reidy et al., 2018).

The objectives of this study were to investigate (1) men's cancer information seeking behaviors, (2) the effect of health literacy on men's cancer information seeking behavior, and (3) men's preferences for cancer information by their level of health literacy. From a national perspective, we were interested in understanding men's information seeking behavior from the Irish Cancer Society (ICS) (www.cancer.ie), the largest provider of cancer information in Ireland.

## Method

### Recruitment

The aim was to recruit men age 40 years and older with varying health literacy levels. Men with inadequate health literacy are difficult to reach (Bonevski et al., 2014). Therefore, a targeted recruitment strategy through the National Adult Literacy Agency (NALA) and Men's Sheds Association (http://menssheds.ie) was employed to reach men with inadequate health literacy. Men's Sheds Association is a nonprofit organization that originated in Australia and has expanded internationally. Individual Men's Sheds work at a community level to advise and improve men's health.

Questionnaire packs (*n* = 635) were dispatched to all NALA literacy classes nationally. An email was sent to the organizers of 250 Irish Men's Sheds Association members asking them to invite members to complete the questionnaire (either via mail or online).

Questionnaire packs contained a questionnaire, information sheet, consent form, return envelope, and pen. Dillman's A tailored design method developed by Dillman, Smyth, & Christian (2009) for maximizing questionnaire response was employed. Other features included use of colored questionnaires, minimizing their length, making it salient, having university endorsement, emphasizing confidentiality, including a postage paid addressed envelope and pen for ease of response (Dillman, Smyth, & Christian, 2009), and placing sociodemographic questions first (Drummond, Sharp, Carsin, Kelleher, & Comber, 2008). Questionnaires were anonymous; no reminders were sent directly to potential participants. A telephone number was given if men needed help completing the questionnaire. Telephone follow-ups were made to organizers of Irish Men's Sheds 3 weeks after mailing, followed by an email reminder after a further 3 weeks.

To reach men with higher health literacy levels, the questionnaire was also available online on SurveyGizmo (www.surveygizmo.com). A dedicated Mens' Cancer Prevention and Health :iteracy study Facebook page and Twitter account were established to distribute the online questionnaire. The study was advertised, and a link to the questionnaire was available on the websites and social media pages of ICS, NALA, Men's Health Forum in Ireland, European Men's Health Forum, and Football Association of Ireland.

### Questionnaire

A cross-sectional questionnaire was developed based on literature review and expert opinion. Measures chosen included key antecedents of information seeking, such as sociodemographic factors, health care access, health status, and opinions of cancer (Friedman, Corwin, Rose & Dominick, 2009; Kelly et al., 2010; Nelissen, Beullens, Lemal & Van den Bulck, 2015). The questionnaire was written in plain English and included 55 questions organized across six sections. The Flesch Reading Ease score was 71.4, equating to an average school grade level of 6.4.

Cancer information seeking was assessed using a screening question adapted from the Health Information National Trends Survey (HINTS) (Nelson et al., 2004): “Have you ever looked for information about cancer from any source?” (*yes*/*no*). Binary outcomes were generated. Men were categorized as “ever” active cancer information seekers if they responded *yes*, and *never* active cancer information seekers if they replied *no*. We asked “Have you ever looked for information from the Irish Cancer Society?” (*yes*/*no*). Men who answered *yes* were categorized as “active,” whereas those who replied *no* were classified as “not active” ICS-specific information seekers. We also asked “Have you ever seen or heard information from the Irish Cancer Society?' (*yes*/*no*). Men were who answered *yes* were categorized as “passive acquirers,” and as “not passive acquirer” of ICS information if they replied *no*.

Health literacy was assessed using a single item, the Brief Screening Questionnaire “How confident are you filling out medical forms by yourself,” scored on a 5-point Likert scale (Chew, Bradley, & Boyko, 2004). This assesses subjective health literacy and was validated against the Rapid Estimate of Adult Literacy in Medicine and Short Test of Functional HL in Adults, (Chew, Bradley, & Boyko, 2004; Chew et al., 2008; Wallace, Rogers, Roskos, Holiday & Weiss, 2006). Men were characterized as having “inadequate” health literacy if they replied *not at all*/*a little*/*somewhat*, and “adequate” health literacy if they replied *quite a bit*/*very confident* (Chew et al., 2004).

Numeric literacy was assessed by asking men to calculate how many 10-mg tablets they would have to take if they required 25 mg daily, categorized as correct (2.5) or not. Disease risk understanding was evaluated by asking “Which of the following represents the biggest risk of getting a disease?” Responses were categorized as correct (1 in 10) or incorrect (1 in 100 or 1 in 1,000).

Men's subjective health rating was measured by asking “How would you describe your current health?” on a 5-point Likert scale (*poor to excellent*) (McDowell, Hughes, & Borrud, 2006). Men's experience of cancer was assessed by asking whether they, their partner, family, or close friends had ever had cancer (*yes*/*no*; Cancer Research UK, 2011).

Men were characterized as having available social support if they *strongly agreed/agreed* with the statement: “I can get access to several people who understand and support me” on a 4-point Likert scale (*strongly agreed* to *strongly disagree*).

Engagement with the health care system was investigated by asking whether men had a general practitioner (GP) (*yes/no*) and to rate their GP's helpfulness on a 4-point Likert scale (*very helpful* to *very unhelpful*). We asked (*yes/no*) whether responders had a medical card (entitles holder to free health care based on income), a GP visit card (entitles holder to free GP visits based on income), or private health insurance.

Cancer fear was assessed with three items from the Cancer Attitude Inventory: (1) “Of all the diseases, I am most afraid of cancer,” (2) “It makes me uncomfortable to think about cancer,” and (3) “I worry a lot about cancer,” using a 5-point Likert scale (*strongly disagree* to *strongly agree* (Berrenberg, 1991). Those who answered *agreed*/*strongly agreed* were classified as having “high cancer fear,” “high discomfort,” and “high cancer worry,” respectively. Otherwise, they were categorized as having “low cancer fear,” “low discomfort,” or “low cancer worry.”

Sociodemographic characteristics collected were age, marital status, nationality, current employment status (employed/not employed), and educational attainment. Educational attainment was categorized as low (no formal education, or up to 10th grade) or high (at least 12th grade or higher level).

Men were asked if they would like to receive cancer information in the future (*yes/no*), and how and where they would like to receive this information.

### Statistical Analysis

We hypothesized that inadequate health literacy negatively affects men's cancer information seeking behavior. Statistical analysis was performed using Stata (v13.0). Chi-squared or Fisher's exact tests were used to investigate factors associated with cancer information seeking. Univariate and multivariate logistic regression models were used to investigate relationships between independent variables known to be correlated with information seeking behavior (sociodemographic factors, current health, access to support, experience of cancer, access to health care, cancer opinions) and health literacy level: (1) “ever” active cancer information seeking, and (2) active seeking, or (3) passive acquisition of ICS information. Independent variables with *p* <.25 in univariate analysis were included in multivariate analyses (Hosmer & Lemeshow, 2000). Tests were two-sided; a score of *p* <.05 was considered statistically significant.

Ethical approval was obtained from the Clinical Research Ethical Committee of the Cork Teaching Hospitals, Ireland.

## Results

Overall, 259 men responded; 164 (62.8%) postal and 95 electronic questionnaires were returned. Significant differences were observed between respondents to postal and online questionnaires (**Table A**).

Mean age of responders was 54 (*SD* = 12), 64% were married, and 51% had low educational attainment (**Table 1**). Current health status was *good/very good/excellent* for 79% of responders, and 73% had access to social support. Almost all had access to a GP (98%) and 84% found them helpful. Of the 259 responders, 41% had private health insurance and 51% had a medical card.

Overall, 44% had inadequate health literacy. About 20% of respondents had a personal cancer diagnosis, and the wife/partner of 9% of men had a prior cancer diagnosis. Cancer fear was high for 61% of men, and 57% reported high discomfort thinking about cancer. High cancer worry was less frequent (31%).

One-half (*n* = 122) of respondents “ever” actively looked for cancer information from any source; 17 men did not answer this question and were excluded from the ever active seeking analyses. A significantly higher proportion of men who “ever” versus “never” actively sought cancer information reported that it is easy to find information (93.3% vs. 75.4%; *p* <.001), and that they compared information from different sources (73.9% vs. 45.9%; *p* <.001), respectively (**Table 1**). No significant differences were observed between “ever” and “never” active cancer information seekers regarding numerical literacy (*p* = 0.281) or disease risk understanding (*p* = 0.498).

In multivariate analysis, odds of “ever” actively seeking cancer information were higher for those who were married (odds ratio [OR] = 2.04; 95% CI [1.08, 3.94]), had higher education (OR = 2.19; 95% CI [1.04, 4.59]), or a personal experience of cancer (OR = 2.73; 95% CI [1.2, 6.21]) (**Table 2**). Health literacy was not significantly associated with ever active cancer information seeking (OR = 1.81; 95% CI [0.90, 3.63]) in multivariate analysis.

Overall, 18.5% (*n* = 48) actively sought, whereas 72.5% (*n* = 171) passively acquired, ICS information (21 respondents did not answer the question on passive acquisition of ICS information and were excluded from the analysis). Having adequate health literacy (OR = 2.93; 95% CI [1.05, 8.15]), being married (OR = 3.42; 95% CI [1.26, 9.24]), and having higher cancer worry (OR = 2.93; 95% CI [1.05, 8.15]) were significantly associated with active ICS cancer information seeking in multivariate analysis.

Odds of passively acquiring ICS information were higher for those who had adequate health literacy (OR = 4.70; 95% CI [1.99, 11.05]), higher education (OR = 2.56; 95% CI [1.01, 6.46]), and who did not find their GP helpful (OR = 0.12; 95% CI [0.02, 0.56]) in multivariate analysis.

**Figure 1.**
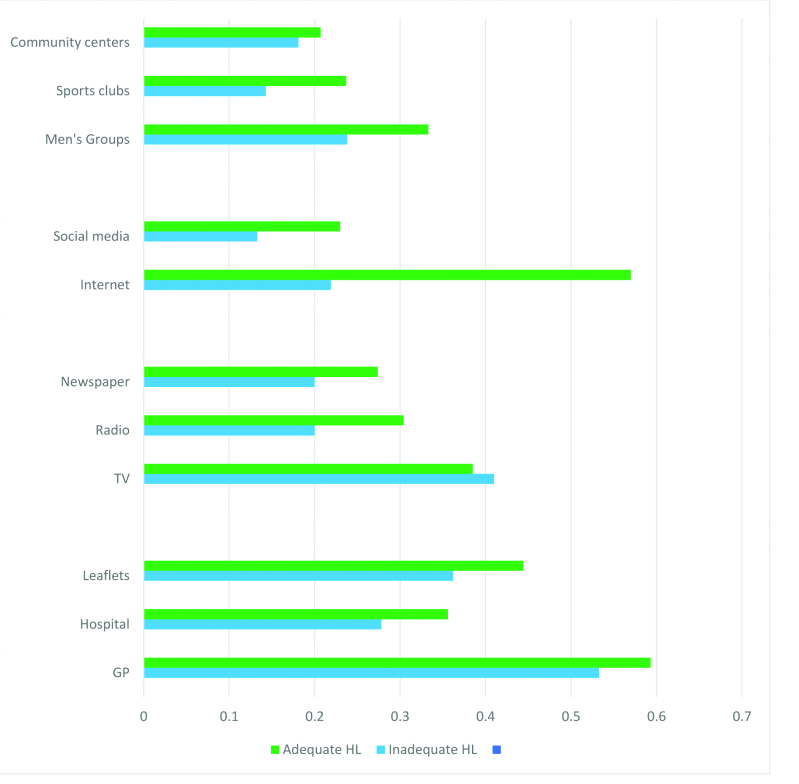
Preferred future cancer information sources for men, stratified by health literacy level. GP = general practitioner; HL = health literacy. Asterisk indicates significant differences (chi-squared *p* value >.05).

The majority (81.4%) want cancer information in the future, with no difference by health literacy level (inadequate 79%, adequate 83%; *p* = .272). Men with adequate versus inadequate health literacy were significantly more likely to want information from every source except from television (**Figure 1**).

GPs were the favored information source for all men (inadequate 53.3%, adequate 59.3%; *p* = 0.215). Men with adequate (vs. inadequate) health literacy would significantly prefer future information on the Internet (57% vs. 21.9%; *p* >.001), social media (23% vs. 13.3%; *p* = .041), and radio (30.4% vs. 20%; *p* = 0.046). Community settings including men's groups were favored by men, more so by those with adequate than inadequate health literacy, with sports clubs (23.7% vs. 14.3%; *p* = .047) being the only one to differ significantly.

## Discussion

This study shows that men age 40 years and older use different cancer information seeking behaviors, with passive information acquisition the most frequently reported. After adjusting for socioeconomic factors, clinical factors, and health care access, health literacy was not correlated with “ever” actively seeking cancer information from any source, but it was significantly correlated with both actively seeking and passively acquiring cancer information from the ICS, a national charity and the biggest producer of cancer information in Ireland. Health literacy level influenced men's preferred future cancer information sources. Findings suggest that a multimodal approach to information format and dissemination is required to reach men with varying health literacy and to reduce gender inequality (i.e., the higher cancer incidence and mortality among men compared with women) (Ferlay et al., 2013).

Health delivery systems endorse self-care and self-management and increasingly place emphasis on people taking responsibility for their health. Therefore, health literacy is an increasingly important concept for researchers, educators, and clinicians to engage with. Health literacy is a life-long process and it is modifiable (Sørensen et al., 2012). This process results in increasing knowledge and skills that enable people to take control of their health, including disease prevention. The first step to adequate health literacy is the ability to seek or acquire health information. Despite this, a meta-narrative systematic review (Saab et al., 2017) that sought to appraise men's cancer information-seeking behaviors identified only three studies (in the years 2006 to 2016) in which literacy and health literacy levels were identified as impediments to information-seeking.

Our findings echo those from other studies. Most men passively acquire, instead of actively seeking, health information in their daily environment and/or from family and friends (Lee et al., 2006; McKenzie, 2003; Niederdeppe et al., 2007). Higher education and being married are correlated with cancer information seeking in this and other studies (Adjei Boakye et al., 2018; Blanch-Hartigan & Viswanath, 2015; Kelly et al., 2010). Levels of cancer fear in this study were similar to those in a large community sample in Britain (Vrinten, van Jaarsveld, Waller, von Wagner, & Wardle, 2014), and in agreement with previous work (Friedman, Corwin, Dominick, & Rose, 2009), and this negatively correlated with men's active cancer information seeking. Access to health care has been shown to correlate with health information seeking (Adjei Boakye et al., 2018). In this study, no such relationship was observed; however, most respondents had access to health care and found their GP helpful. Additionally, those who found their GP helpful reported passively acquiring ICS information less often, possibly because they were satisfied with the information from their GP.

In this study, a higher proportion of men with cancer, compared to those without, actively looked for cancer information. This contrasts with the findings of Adjei Boakye et al. (2018); however, the present study was smaller and used a purposeful recruitment strategy, which may explain these differences. When we adjusted for these factors, those with inadequate health literacy had lower odds of seeking or acquiring cancer prevention information from the ICS. Reasons why these men do not actively seek or passively acquire cancer prevention information remain largely unknown and may be related to factors not measured in this study. In the health literacy concept model, as well as access, perception and use of cancer information also differ with health literacy level (Best et al., 2018; Shim, Kelly & Hornik, 2006; Smith, Trevena, Nutbeam, Barratt, & McCaffery, 2008). A study from the U.S. using Health Information National Trends data found that people with low health literacy were more likely to hold fatalistic cancer prevention beliefs (Fleary, Paasche-Orlow, Joseph, & Freund, 2018). This in turn could inhibit men with inadequate health literacy from seeking or acquiring any cancer information; however, more research is required.

Men of all health literacy levels want cancer information in a format they can understand and want it from multiple places. GPs are the preferred source for men of all health literacy levels in Ireland, followed by the Internet for men with adequate health literacy and television for men with inadequate health literacy. GPs are increasingly busy; therefore, different information sources are being used by organizations and people. An increased understanding of the implications of inadequate health literacy has resulted in more attention being paid to the readability of printed materials. However, the simultaneous increase in health information dissemination on the Internet negates this advancement to some extent because of the high literacy, numeracy, and computer skills required to navigate health-related websites. Increased Internet use for cancer information by older men and non-White groups has been described in the U.S. (Huerta, Walker, Johnson, & Ford, 2016). However, dissemination of cancer information on the Internet and television can result in increased cancer fear, which in turn influences cancer information seeking (Nelissen et al., 2015). Other modes of health information dissemination, such as Men's Sheds, have been shown to be effective and could be used more extensively, especially for those with inadequate health literacy (Misan, Oosterbroek, & Wilson, 2017).

## Study Limitations

This study has several limitations. First, targeted recruitment strategies used may have had an effect on the general-izability of the findings. Second, the study used two survey formats (paper, online). Third, the study used one validated screening question as the health literacy measure, and one cancer information seeking measure. Finally, we have no information on nonresponders or reasons for nonresponse.

## Implications

Those developing health information need to make cancer information simpler, more accessible, and more meaningful for adults with inadequate health literacy. Because of the numerous things now competing for our attention in daily life, incidental information acquisition is the reality for most people. Short, easy-to-digest, “bite-sized” information is more likely to be transmitted to larger numbers with inadequate health literacy. Furthermore, multimodal dissemination has been successfully employed (Friedman et al. 2009; Simmons et al., 2017). Finally, GPs need to be able to identify those with inadequate health literacy and to be supported in their role as important sources of cancer prevention information, especially for this vulnerable group.

## Conclusions

Health literacy is correlated with active seeking and passively acquiring cancer information in a non–health care context. To reach men with inadequate health literacy, dissemination of cancer information in simple and multimodal formats is required. This may improve men's cancer prevention information seeking, and reduce gender inequities and men's cancer burden.

## Figures and Tables

**Table 1:** Characteristics of Responders by “Ever” Cancer Information Seekers, Active Irish Cancer Society Information Seekers, and Passive Acquisition of Irish Cancer Society Information

**Characteristic**	**“Ever” Cancer Information Seeker**				**Active ICS Information Seeker**			**Passive ICS Information Gatherer**		
		
	**Total, *n* (%)**	**Ever Active Cancer Information Seeker, *n* (%)**	**Never Active Cancer Information Seeker, *n* (%)**	***p* Value^a^**	**Active ICS Information Seeker, *n* (%)**	**Not Active ICS Information Seeker, *n* (%)**	***p* Value^a^**	**Passive ICS Information Acquirer,^b^*n* (%)**	**Not Passive ICS Information Acquirer, *n* (%)**	***p* Value^a^**

Total	242 (100)	122 (49.8)	122 (50.2)		48 (17.3)	211 (82.7)		171 (72.5)	65 (27.5)	

Sociodemographic factor										

Marital status										
Married/living as married	**153 (64.6)**	**85 (72)**	**68 (57.1)**		**34 (77.3)**	**133 (63.3)**		**119 (69.6)**	**32 (49.2)**	
Other	**84 (35.4)**	**33 (28)**	**51 (42.9)**	**.017**	**10 (22.7)**	**77 (36.7)**	**.083**	**52 (30.4)**	**33 (50.8)**	**.004**
Age group										
<50 years	85 (35.9)	38 (31.9)	47 (39.8)	.478	16 (35.6)	81 (38.8)	.194	62 (36.3)	22 (33.8)	.944
50–59 years	76 (32.1)	40 (33.6)	36 (30.5)		13 (28.9)	68 (32.5)		55 (32.2)	21 (32.3)	
60–69 years	58 (24.5)	33 (27.7)	25 (21.2)		15 (33.3)	43 (20.6)		42 (24.6)	16 (24.6)	
≥70 years	18 (7.6)	8 (6.7)	10 (8.5)		1 (2.2)	17 (8.1)		12 (7)	6 (9.2)	
Education status										
Low	**123 (51)**	**47 (38.8)**	**76 (63.3)**	**<.001**	**15 (33.3)**	**112 (52.6)**	**.022**	**74 (42.5)**	**47 (71.2)**	**<.001**
High	**118 (49)**	**74 (61.2)**	**44 (36.7)**		**30 (66.7)**	**101 (47.4)**		**100 (57.5)**	**19 (28.8)**	

Health literacy										

Health literacy level										
Inadequate	**105 (43.9)**	**37 (31.1)**	**68 (56.7)**		**10 (22.7)**	**95 (48.5)**		**54 (31.4)**	49 (74.2)	**<.001**
Adequate	**134 (56.1)**	**82 (68.9)**	**52 (43.3)**	**<.001**	**34 (77.3)**	**101 (51.5)**	.002	**118 (68.6)**	19 (25.8)	

Health and support										

Current health status										
Poor/fair	50 (20.8)	28 (23.3)	22 (18.3)	.340	12 (27.3)	40 (19.3)	.305	35 (20.3)	15 (22.4)	.728
Good/very good/excellent	190 (79.2)	92 (79.7)	98 (81.7)		32 (72.7)	167 (80.7)		137 (79.7)	52 (77.6)	
Access to support?										
Strongly agree/agree	173 (73.3)	89 (74.8)	84 (71.8)		32 (74.4)	142 (73.2)		128 (75.3)	44 (67.7)	
Strongly disagree/disagree	63 (26.7)	30 (25.2)	33 (28.2)	.603	11 (25.6)	52 (26.8)	1	42 (24.7)	21 (32.3)	.239

Experience of cancer										

Personal cancer diagnosis										
No	**193 (80.1)**	**88 (72.1)**	**105 (88.2)**	**.002**	**28 (62.2)**	**168 (82)**	**.005**	138 (79.3)	55 (82.1)	.628
Yes	**48 (19.9)**	**34 (27.9)**	**14 (11.8)**		**17 (37.8)**	**37 (18)**		36 (20.7)	12 (17.9)	
Partner with cancer										
No	**219 (91.3)**	**106 (86.9)**	**113 (95.8)**	**.015**	39 (86.7)	189 (92.2)	.247	155 (89.1)	64 (97)	.053
Yes	**21 (8.8)**	**16 (13.1)**	**5 (4.2)**		6 (13.3)	16 (7.8)		19 (10.9)	2 (3)	
Family member/friend with cancer										
No	38 (15.8)	18 (14.8)	20 (16.8)	.662	7 (15.6)	32 (15.5)	1	27 (15.5)	12 (17.9)	.651
Yes	203 (84.2)	104 (85.2)	99 (83.2)		39 (84.4)	174 (84.5)		147 (84.5)	55 (82.1)	

Access to health care										

Private health insurance										
No	144 (59.5)	67 (54.9)	77 (64.2)	.143	23 (51.1)	126 (58.9)	.407	98 (56.3)	45 (67.2)	.125
Yes	98 (40.5)	55 (45.1)	43 (35.8)		22 (48.9)	88 (41.1)		76 (43.7)	22 (32.8)	
Has a GP?										
No	4 (1.7)	2 (1.7)	2 (4.5)	1^c^	1 (2.2)	10 (4.8)	.695	1 (0.6)	3 (4.5)	.0672
Yes	237 (98.3)	119 (98.3)	118 (98.8)		44 (97.8)	197 (95.2)		172 (99.4)	64 (95.5)	
Finds GP helpful?				.780						
No	36 (15.3)	19 (16)	17 (14.7)		8 (17.8)	32 (15.9)	.823	**35 (20.5)**	**2 (3.2)**	**.001**
Yes	199 (84.7)	100 (84)	99 (85.3)		37 (82.2)	169 (84.1)		**136 (79.5)**	**61 (96.8)**	

Opinions about cancer										

Cancer fear										
Lower fear	92 (39.1)	52 (44.4)	40 (33.9)	.098	18 (40)	79 (39.7)	1	68 (41)	24 (35.8)	.467
Higher fear	143 (60.9)	65 (55.6)	78 (66.1)		27 (60)	120 (60.3)		98 (59)	43 (64.2)	
Cancer discomfort										
Lower discomfort	101 (43)	**64 (54.7)**	**37 (31.4)**	**<.001**	19 (43.2)	87 (43.5)	1	**80 (48.2)**	**21 (31.3)**	**.019**
Higher discomfort	132 (56.7)	**86 (51.8)**	**46 (68.7)**		25 (56.8)	113 (56.5)		**86 (51.8)**	**46 (68.7)**	
Cancer worry										
Lower worry	158 (68.7)	83 (72.2)	75 (65.2)	.255	25 (58.1)	140 (71.8)	.100	117 (72.2)	41 (62.1)	.134
Higher worry	72 (31.3)	32 (27.8)	40 (34.8)		18 (41.9)	55 (28.2)		45 (27.8)	25 (37.9)	

Information seeking factors										

Difficulty understanding written material										
No	**192 (80.7)**	**103 (85.8)**	**89 (75.4)**	**.042**	**44 (95.6)**	**147 (77)**	**<.001**	**146 (84.9)**	**45 (69.2)**	**.007**
Yes	**46 (19.3)**	**17 (14.2)**	**29 (24.6)**		**2 (4.3)**	**44 (23)**		**26 (15.1)**	**20 (30.8)**	
Help with understanding health information										
No	186 (80.2)	98 (85.2)	88 (75.2)	.056	**41 (91.1)**	**145 (76.7)**	**<.001**	**140 (84.3)**	**46 (71.9)**	**.031**
Yes	46 (19.8)	17 (14.8)	29 (24.8)		2 (8.8)	**44 (21.7)**		**26 (15.7)**	**18 (28.1)**	
Confident working out tablet dose										
No	47 (19.9)	19 (16.4)	28 (23.3)	.181	11 (23.9)	50 (26.3)	.668	**27 (16.1)**	**20 (29.9)**	**.017**
Yes	189 (80.1)	97 (83.6)	92 (76.7)		35 (76.1)	140 (73.7)		**141 (83.9)**	**47 (70.1)**	
Calculated correct tablet dose										
No	61 (25.7)	27 (22.7)	34 (28.8)	.281	9 (20)	38 (20)	.779	42 (24.6)	19 (29.2)	.464
Yes	176 (74.3)	92 (77.3)	84 (71.2)		36 (80)	152 (80)		129 (75.4)	46 (70.8)	
Correct disease risk										
No	60 (25.9)	32 (27.8)	28 (23.9)	.498	37 (84.1)	134 (71.7)	.179	40 (23.8)	21 (33.3)	.144
Yes	172 (74.1)	83 (72.2)	89 (76.1)		7 (15.9)	53 (28.3)		128 (76.2)	42 (66.7)	
Easy to find information										
No	**36 (15.4)**	**8 (6.7)**	**28 (24.6)**	**<.001**	4 (8.7)	31 (16.6)	.162	**18 (10.5)**	**16 (26.2)**	**.003**
Yes	**198 (84.6)**	**112 (93.3)**	**86 (75.4)**		42 (91.3)	156 (83.4)		**154 (89.5)**	**45 (73.8)**	
Compares health information from different sources										
No	**91 (39.6)**	**31 (26.1)**	**60 (54.1)**	**<.001**	12 (25.5)	78 (43.1)	.086	**52 (31.1)**	**37 (60.7)**	**<.001**
Yes	**139 (60.4)**	**88 (73.9)**	**51 (45.9)**		35 (74.5)	103 (56.9)		**115 (68.9)**	**24 (39.3)**	

Note. Bold text indicates statistical significance. Missing values are not included in the table. GP = general practitioner; ICS = Irish Cancer Society.

a Chi-squared test.

b 21 respondents did not answer this question and were eliminated from the analysis.

c Fisher's exact test.

**Table 2:** Multivariate Logistic Regression Analyses of Factors Significantly Associated with Ever Cancer Information Seeking, Active ICS Information Seeking (*N* = 203), and Passive Acquisition of ICS Information

**Characteristic**	**Ever Cancer Information Seeker^a^**		**Active ICS Information Seeker^b^**		**Passive ICS Information Acquirer^c^**	
		
	**OR [95% CI]**	***p* Value**	**OR [95% CI]**	***p* Value**	**OR [95% CI]**	***p* Value**

Sociodemographic factors						

Marital status						
Other	**1**		**1**		1	
Married/living as married	**2.04 [1.06, 3.94]**	**.034**	**3.42 [1.26, 9.24]**	**.016**	2.08 [0.98, 4.42]	.058
Education status						
Low	**1**		1		**1**	
High	**2.19 [1.04, 4.59]**	**.039**	2.46 [0.87, 6.93]	.089	**2.56 [1.01, 6.46]**	**.047**

Health literacy						

Health literacy level						
Inadequate	1		**1**		**1**	
Adequate	1.81 [0.90, 3.63]	.097	**2.93 [1.05, 8.15]**	**.039**	**4.7 [1.99, 11.05**]	**<.001**

Experience of cancer						

Personal cancer diagnosis						
No	**1**		1		-	-
Yes	**2.73 [1.2, 6.21]**	**.016**	2.08 [0.87, 4.97]	.099		

Access to health care						

Finds GP helpful?						
No	-	-	-	-	**1**	
Yes					**0.12 [0.02, 0.56]**	**.007**

Opinions about cancer						

Cancer discomfort						
Lower	1		-	-	1	
Higher	0.52 [0.25, 1.09]	.083			0.83 [0.35, 1.97]	.674

Cancer worry						
Lower	1		1		1	
Higher	1.2 [0.53, 2.72]	.097	**3.68 [1.39, 9.79]**	**.009**	1.08 [0.45, 2.6]	0.863

Note. Bold text indicates statistical significance. CI = confidence interval; GP = general practitioner; ICS = Irish Cancer Society; OR = odds ratio.

a Multivariate model included martial status, age, educational attainment, health literacy level, personal cancer diagnosis, cancer discomfort, cancer fear, cancer worry, and partner with cancer.

b Multivariate model included martial status, educational attainment, health literacy level, personal cancer diagnosis, private health insurance status, and cancer worry.

c Multivariate model included martial status, educational attainment, health literacy level, private health insurance status, finds general practitioner helpful, cancer discomfort, partner with cancer, access to support, and cancer worry.

**Table A:** Characteristics of All Responders, Stratified by Mode of Response (Postal and Online)

**Characteristic**	**Total, *N* (%)** **259 (100)**	**Online, *n* (%)** **95 (37)**	**Postal, *n* (%)** **164 (63)**	***p* Value^a^**

Marital status				
Married/living as married	**167 (65.7)**	**69 (73)**	**94 (59)**	
Other	**87 (34.3)**	**24 (25)**	**62 (39)**	**.021**
Age group				
<54 years	**134 (52)**	**68 (71.6)**	**66 (40.2)**	**<.001**
≥54 years	**120 (47)**	**27 (28.4)**	**93 (56.7)**	
Education status				
Low	**127 (49.2)**	**19 (20)**	**108 (66.3)**	**<.001**
High	**131 (50.8)**	**76 (80)**	**55 (35)**	

Health literacy level				
Inadequate	**105 (43.8)**	**15 (19)**	**90 (55.9)**	**<.001**
Adequate	**135 (56.3)**	**64 (81)**	**71 (44.1)**	

Current health status				
Poor/fair	52 (20.7)	14 (16.1)	38 (23.2)	.124
Good/very good/excellent	199 (79.3)	73 (83.9)	126 (76.8)	
Access to support?				
Strongly agree/agree	174 (73.4)	49 (62.8)	125 (78.6)	
Strongly disagree/disagree	63 (26.6)	29 (37.2)	34 (21.4)	.008

Personal cancer diagnosis				
No	**197 (78.5)**	**50 (56.8)**	**147 (90.2)**	**<.001**
Yes	**54 (21.5)**	**38 (43.2)**	**16 (9.8)**	
Partner with cancer				
No	228 (91.2)	78 (88.6)	150 (92.6)	.204
Yes	22 (8.8)	10 (11.4)	12 (7.4)	
Family member/friend with cancer				
No	39 (15.5)	8 (9.1)	31 (19.0)	.027
Yes	212 (84.5)	80 (90.9)	132 (81.0)	

Private Health Insurance				
No	149 (57.5)	34 (35.8)	115 (70.1)	**<.001**
Yes	110 (42.5)	61 (64.2)	49 (29.9)	
Has a GP?				
No	11 (4.4)	5 (5.7)	6 (3.7)	.327
Yes	241 (95.6)	83 (94.3)	158 (96.3)	
Finds GP helpful?				
No	40 (16.3)	13 (14.8)	27 (17.1)	.390
Yes	206 (83.7)	75 (85.2)	131 (82.9)	
Cancer fear				
Lower fear	97 (39.8)	48 (55.2)	49 (31.2)	<.001
Higher fear	147 (60.2)	39 (44.8)	108 (68.8)	
Cancer discomfort				
Lower discomfort	106 (43.4)	**52 (59.8)**	**54 (34.4)**	<.001
Higher discomfort	138 (56.6)	**35 (40.2)**	**103 (65.6)**	
Cancer worry				
Lower worry	165 (69.3)	65 (77.4)	100 (64.9)	.031
Higher worry	73 (30.7)	19 (22.6)	54 (35.1)	

Information seeking factors				

Difficulty understanding written material				
No	**192 (80.7)**	**103 (85.8)**	**89 (75.4)**	**.042**
Yes	**46 (19.3)**	**17 (14.2)**	**29 (24.6)**	
Help with understanding health information				
No	186 (80.2)	98 (85.2)	88 (75.2)	.056
Yes	46 (19.8)	17 (14.8)	29 (24.8)	
Confident working out tablet dose				
No	47 (19.9)	19 (16.4)	28 (23.3)	.181
Yes	189 (80.1)	97 (83.6)	92 (76.7)	
Calculated correct tablet dose				
No	61 (25.7)	27 (22.7)	34 (28.8)	.281
Yes	176 (74.3)	92 (77.3)	84 (71.2)	
Correct disease risk				
No	60 (25.9)	32 (27.8)	28 (23.9)	.498
Yes	172 (74.1)	83 (72.2)	89 (76.1)	
Easy to find information				
No	**36 (15.4)**	**8 (6.7)**	**28 (24.6)**	**<.001**
Yes	**198 (84.6)**	**112 (93.3)**	**86 (75.4)**	
Compares health information from different sources				
No	**91 (39.6)**	**31 (26.1)**	**60 (54.1)**	**<.001**
Yes	**139 (60.4)**	**88 (73.9)**	**51 (45.9)**	
Active “ever” information seeker from any source				
No	**120 (49.6)**	**22 (27.8)**	**98 (60.1)**	**<.001**
Yes	**122 (50.4)**	**57 (72.2)**	**65 (39.9)**	

Note. Bold text indicates statistical significance. Missing values are not included in the table.

a Chi-squared test or Fisher's exact test.
